# Appropriate Prescribing for older adults with Multimorbidity (Pro-M): protocol for a feasibility study

**DOI:** 10.1186/s13690-024-01264-x

**Published:** 2024-03-18

**Authors:** Jia Ying Tang, Poh Hoon June Teng, Christine Yuanxin Chen, Keng Teng Tan, Wendy Ang, Sabrina Lau, Alexis Guat Cheng Ang, Kay Khine Kyaw, Xin Yong Tay, Wan Min Stephanie Lim, Wrenzie Del Valle Espeleta, Huimin Lin, Yew Yoong Ding, Penny Lun

**Affiliations:** 1 Geriatric Education & Research Institute, 2 Yishun Central 2, Singapore, 768024 Singapore; 2 Department of Geriatric Medicine, Changi General Hospital, Singapore, Singapore; 3 Pharmacy, Tan Tock Seng Hospital, Singapore, Singapore; 4 Pharmacy, Changi General Hospital, Singapore, Singapore; 5 Department of Geriatric Medicine, Tan Tock Seng Hospital, Singapore, Singapore

**Keywords:** Feasibility study, Potentially inappropriate prescribing, Older adults, Proctor’s implementation outcomes, Medication review

## Abstract

**Background:**

Potentially inappropriate prescribing is common among older adults with multimorbidity due to various reasons, from concurrent application of multiple single-disease clinical guidelines to fragmentation of care. Interventions such as medication review have been implemented worldwide to reduce inappropriate prescribing for older adults. However, the implementability of such interventions are underexplored in the outpatient clinics in Singapore’s public hospitals. Hence, the Pro-M study aims to assess the feasibility of implementing a physician-pharmacist collaborative care intervention in geriatric medicine outpatient clinics to facilitate appropriate prescribing for older adults in Singapore.

**Methods:**

This is a single-arm, non-randomised feasibility study using a pre-post evaluation design. This study consists of two parts: (1) implementation phase of the intervention (6 months) and an (2) evaluation phase (3 months). Eligible patients will be recruited from geriatric medicine outpatient clinics at two public hospitals in Singapore through convenience sampling. The main components of the Pro-M intervention are: (1) pharmacist-facilitated medication reviews with feedback on any medication issues and potential recommendations to physicians, and (2) physicians communicating changes to other relevant prescribers. The evaluation phase will involve surveying and interviewing physicians and pharmacists involved in the implementation of the intervention. A mixed-method approach will be employed for data collection and analysis. The quantitative and qualitative findings will be triangulated and reported using Proctor’s implementation outcomes: appropriateness, penetration, acceptability, fidelity, feasibility, and sustainability. A basic cost analysis will be conducted alongside the study.

**Discussion:**

This is a phase 2 study to test the feasibility of implementing an intervention that was co-created with stakeholders during phase 1 development of an intervention to optimise prescribing for older adults with multimorbidity. The implementation will be assessed using Proctor’s implementation outcomes to provide insights on the process and the feasibility of implementing medication reviews for older adults with multimorbidity as a routine practice in outpatient clinics. Data collected from this study will inform a subsequent scale-up study.

**Trial registration:**

ClinicalTrials.gov Identifier: NCT05756478. Registered on 06 March 2023.

**Supplementary Information:**

The online version contains supplementary material available at 10.1186/s13690-024-01264-x.

**Table:** 

**Text box 1. Contributions to the literature**
• The Pro-M study aims to assess the feasibility of implementing medication review for older adults with multimorbidity in a busy clinical setting such as the geriatric medicine outpatient clinics in Singapore.
• This study would observe the direction of the effects of a physician-pharmacist collaborative care intervention in reducing potentially inappropriate medications in older adults attending hospital-based geriatric medicine clinics.
• The results will inform future plans to scale up the Pro-M intervention in other outpatient specialists’ clinics in Singapore.

## Introduction

An ageing population is a rising concern around the world. In 2022, 18.4% of Singapore’s population were aged 65 and above, and this number is predicted to increase to 23.8% in 2030 [1]. Among older adults, multimorbidity of having at least two or more chronic conditions is a common occurrence [2, 3] and in Singapore, one study identified 51.5% of older adults aged 60 and above to have multimorbidity [4]. Multimorbidity is often associated with polypharmacy, which is often defined in literature as taking five or more medications concurrently [5]. Polypharmacy increases one’s risk of experiencing negative clinical outcomes, such as adverse drug reactions (ADRs), falls, and hospitalization [6]. It also increases the likelihood of potentially inappropriate prescribing (PIP) [7]. One of the reasons for the increased risk is that most clinical guidelines and evidence for disease management are focused on treating a single disease [8]. However, polypharmacy can be considered appropriate when the medications prescribed are in line with best evidence [8]. Therefore, being prescribed appropriate medications is more critical, regardless of the numbers [9]. Nonetheless, prescribing for older adults with multimorbidity can be a complex task with the need to factor in various diagnoses as well as the decline in their physiological conditions [10, 11].

Interventions to address PIP among older adults have been conducted worldwide, spanning from pharmacist-related interventions, education, and the involvement of a multidisciplinary team [12–14]. Prescribing tools such as Beers criteria [15], Screening Tool of Older People’s Prescriptions (STOPP) criteria, and Screening Tool to Alert to Right Treatment (START) criteria [16] have also been used to assess inappropriate medication use in older adults [13]. A scoping review has identified medication review as an element in almost 70% of the interventions aimed at reducing PIP among older adults, with an average of 2.5 elements per intervention [17]. Additionally, a review by Bloomfield et al. (2020) assessed the effectiveness of deprescribing interventions for community-dwelling older adults and found that comprehensive medication review may have reduced potentially inappropriate medications (PIMs), along with a slight reduction in mortality [18].

In a Singapore study conducted in 2019, the prevalence of polypharmacy in community-dwelling older adults in Singapore was found to be around 14.5% and is correlated with medication non-adherence [19]. Other factors, such as age (85 years and above), gender (male), health conditions, and ethnicity (Malay or Indian) also increase one’s likelihood of having polypharmacy [19]. Similarly, 58.6% of nursing home residents were exposed to polypharmacy, with PIP observed in 70% of the residents [20]. There have also been efforts to optimise prescribing for older adults in Singapore. A local study done in the nursing homes setting found pharmacist medication reviews beneficial and improve the quality of life and care for the residents indirectly [21]. Another study conducted in the inpatient setting using an implicit tool, recommended its use to review and identify inappropriate medications in a busy setting [22]. However, medication reviews are not part of routine practice in the busy outpatient clinics, due to the time and coordination needed. Hence, the feasibility and effectiveness of implementing such an intervention in the outpatient setting is unknown.

Since medication review in settings like the outpatient clinics has not been well-studied, we embarked on a phase 1 intervention-development study, using theory- and evidence-based, implementation-based, and partnerships approaches to co-create an intervention with relevant stakeholders to address potential contextual challenges. Details on the intervention development process was published in a separate paper [23].

The resulting intervention, Pro-M or Appropriate Prescribing for older adults with Multimorbidity, is a physician-pharmacist collaborative care intervention to optimise prescribing for older adults with multimorbidity at outpatient specialist clinics in Singapore’s public hospitals. The main component is a pharmacist-facilitated medication review, with physicians communicating changes to other relevant prescribers. This second phase study aims to evaluate the feasibility of the intervention from the perspectives of the physicians and pharmacists involved in the implementation. The rationale of conducting a feasibility study is to test and evaluate the implementation of the intervention in real-world settings and identify potential implementation challenges that need to be addressed and adapted for the full-scale study in order to reduce potential resource wastage [24, 25]. As the intervention was co-created with stakeholders from the two study sites, our conjecture is that the implementation of the intervention would likely be feasible to the physicians and pharmacist. In addition, our study aims to provide quality care to the older population in Singapore, which aligns with the Singapore’s Voluntary National Review [26] which takes guidance from United Nations’ Sustainable Development Goals (SDGs) [27] on the goal of good health and well-being.

### Study aims and objectives

The aims of the study are:

1. To assess feasibility of the intervention among stakeholders using Proctor’s implementation outcomes [28]: Appropriateness, Penetration, Acceptability, Fidelity, Feasibility, and Sustainability (primary).
2. To collect data on recruitment rate for sample size calculation in the next phase scale-up study (secondary).
3. To collect pre-post data on the prevalence of PIMs and/or other medication issues for sample size calculation in the next phase scale-up study (secondary).
4. To conduct a cost analysis of the intervention based on manpower cost and the cost of PIMs and/or medications with other issues identified before and after the medication reviews (secondary).

## Methods

### Study design

**Fig. 1:**
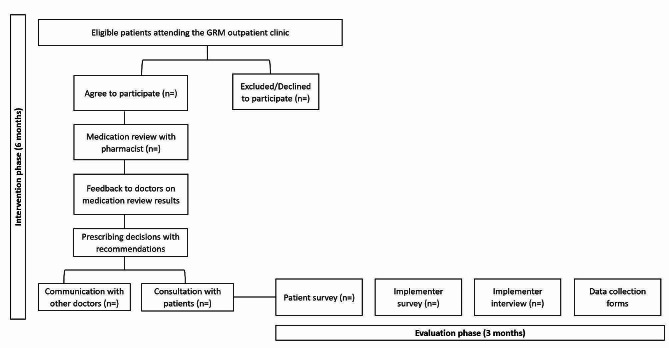
Overview of the Pro-M study design

This is a single-arm, non-randomised feasibility study using a pre-post evaluation design. Any medication changes related to PIM and other medication issues before and after medication review will be compared. An explanatory sequential mixed method approach will be used for data collection and analysis, where the qualitative findings (e.g., interviews) will be used to explain the quantitative findings (e.g., recruitment rate, implementer survey results) [29]. This study consists of two parts: (1) implementation phase of the intervention (6 months) and an (2) evaluation phase (3 months). An outline of the feasibility study including the intervention and evaluation phases is shown in Fig. 1. Ethics approval was obtained from National Healthcare Group Domain Specific Review Board (NHG DSRB) domain F (Ref. no: 2022/00491). The reporting of this protocol is guided by the standard protocol items: recommendations for interventional trials (SPIRIT) [30] (see Additional file 1: SPIRIT 2013 checklist). The Clinical Trial registration for this study is NCT05756478.

### Setting

The study will be conducted at the geriatric medicine (GRM) outpatient clinic of two public acute hospitals in Singapore. In Singapore, 80% of the primary care is provided by the private sector but the opposite is observed for secondary care (e.g., outpatient settings) [31, 32]. As our study target older adults, GRM outpatient clinics providing care to older patients with multiple chronic conditions are the ideal location to trial the intervention. Furthermore, the intervention was co-created with the stakeholders from both study sites and that medication review for older adults with multimorbidity is not a routine practice. The clinic physicians are invited to refer eligible patients to the study, while the pharmacists conducting medication reviews are part of the study teams at each hospital site.

### Screening and recruitment of patients

Eligible patients will be pre-screened and invited to join the study by their attending GRM physicians through convenience sampling. The inclusion criteria are as follow: aged 65 and above, current patient of GRM outpatient clinic, and taking five or more medications daily. On the other hand, patients will be excluded if they are below 65 years old, currently receiving other types of pharmacist services (e.g., medication therapy management), are unable to understand and communicate in English, Chinese, or Malay and if patient or caregiver decline to participate in the study. Participation in this study is fully voluntary, and if they agree to participate, written informed consent will be taken face-to-face by a study team member at each hospital. There is no consensus on the optimal sample size for pilot or feasibility studies and the size is dependent on the objective of the study [33]. Hence, we did not perform a sample size calculation. Instead, we consulted and discussed with stakeholders at both sites and agreed to recruit 30 patients per site for this study, which will provide sufficient insights into the implementation process.

### Intervention specification

The prototype for the intervention evolved from multiple scoping reviews and modified Delphi studies and was finalised through a co-creation exercise with stakeholders consisting of geriatricians and pharmacists at both study sites during phase 1. In summary, various theoretical frameworks and taxonomy, such as Theoretical Domains Framework (TDF), Behaviour Change Wheel (BCW), and Behaviour Change Techniques (BCTs), were employed at different time points to develop the intervention [34–36]. The detailed account of the intervention development process has been reported in another publication [23]. The main components for this intervention are: (1) pharmacist-facilitated medication review with feedback and recommendation to physicians and (2) physicians communicating medication changes made by other prescribers when needed. Table 1 shows the process of operationalisation of the BCTs in the Pro-M study.

**Table 1:** Operationalisation of BCTs in Pro-M study

BCTs identified from modified Delphi study [37]	Context during phase 1 prototype development	Operationalizing BCTs in the Pro-M Study
Credible source	Pharmacists who are experienced in geriatric pharmacology	Medication reviews are led by pharmacists, who will be present at the clinic during review sessions
Restructuring the physical environment	Presence of pharmacist in clinics	
Instruction on how to perform the behaviour	Guidelines to assist in optimizing prescribing	Pharmacists will conduct medication review using prescribing tools (e.g., Beers Criteria)
Information about health consequences	Feedback from pharmacists on occurrence of PIMs post-medication review	Physicians will receive patient-specific feedback on PIMs or other medication issues identified by pharmacists
Feedback on outcomes of behaviour		
Problem solving	Physicians will identify medication problems and/or discrepancies, and then discussing with pharmacists as well as patients for necessary changes to be made	Physicians will ascertain medication review outcomes and any recommendations from the pharmacists, before making the next prescribing decision (e.g., to reduce PIMs)
Goal setting (outcome)		
Goal setting (outcome)	Ensuring proper documentation on reasons behind addition or removal of medications and provide feedback to other relevant prescribers	Documentation of medication indications and communicating changes to other relevant prescribers
Feedback on outcomes of behaviour		

As our intervention was developed with the considerations on integrating medication review into routine outpatient care, variations in the delivery format between the sites were accommodated, due to the operational context and preference in practice at each site. For instance, there are two modes of recruiting eligible patients at both sites. One site has the option of calling patients beforehand to introduce the study, whereas the other site will mainly recruit patients on-the-spot during the day of appointment. Patients who consent to participate in the study will undergo a one-time medication review with a pharmacist. Medication review will be conducted either in-person or through a tele-med consultation, using preferred prescribing tools of choice (e.g., Beers criteria, STOPP/START) by the site pharmacists. Findings of any PIMs and other issues will be highlighted to the physicians so that they could take this information into consideration when making prescribing decisions during their consultations with patients. The changes would then be documented and communicated to the patients and other relevant prescribers via discussions or a memo. The feasibility of implementing medication reviews in routine outpatient practice among the stakeholders (patients, physicians, pharmacists) will be evaluated through surveys. In addition, in-depth interviews will also be conducted with selected implementers (physicians and pharmacists) to understand additional barriers experienced in the implementation process.

### Outcome measures

#### Primary outcome and data collection

The primary focus of this study is to assess the feasibility of implementing the physician-pharmacist collaborative care intervention from the stakeholders’ perspectives, which will be reported qualitatively. Surveys will be conducted to explore stakeholders’ attitudes and experiences during the intervention. In-depth interviews will follow with selected implementers to elicit insights as well as qualitative interpretations to the survey findings.

A short 11-item patient survey (see Additional file 2: Pro-M patient survey) will be administered after the intervention to measure patients’ attitudes toward acceptance and appropriateness of the intervention. They will also be asked if they will be willing to pay for medication review as part of their routine care in the future. On the other hand, physicians and pharmacists involved in the intervention will be invited to participate in the evaluation phase. To achieve this goal, a 25-item implementer survey was developed using Proctor’s implementation framework (Penetration, Appropriateness, Acceptability, Fidelity, Feasibility, and Sustainability) [28] and each item will be measured using a 5-point Likert scale (see Additional file 3: Pro-M implementer survey).

In addition, some quantitative data, such as time taken to conduct the medication review, number of discussions between physicians and pharmacists, number of communication efforts by the physicians, and the number of agreements between the physicians and the pharmacists on PIM and other medication issues identified, will be collected alongside the intervention. This quantitative information will be triangulated with the survey and interview results to provide a fuller picture on the feasibility of the intervention.

#### Secondary outcomes and data collection

To provide information for sample size calculation for the next phase scale-up study, the average number of PIMs per patient before and after medication review and the prevalence of PIMs among patients will be collected. For the purpose of this study, prevalence is defined as having at least one PIM prescribed. Medication data on identified PIM and other medication issues will be collected over two time points: once during the medication review and a retrospective data collection from patient’s last GRM appointment. The retrospective data will form the comparison and represent ‘usual care’ where medication review is only done in an ad-hoc and informal basis, if any. This comparison will help to ascertain if medication reviews impact physicians’ prescribing decisions in any way.

In addition, a basic cost analysis will be conducted to compare the cost of manpower for medication review and the cost of PIMs or other medications issues identified before and after the medication reviews. Manpower cost to conduct medication reviews will be estimated by the time needed to a conduct a medication review. Cost of PIMs and other medication issues identified and resolved will be collected to estimate the monthly cost savings from discontinued medications and any substituted medications, if there are [21]. The unit cost of medications will be calculated using the private costs of medications before government subsidies. The cost information collected will provide insights on the longer-term sustainability of implementing medication review in routine care for older adults with multimorbidity.

### Data analysis

#### Quantitative data

Descriptive statistics will be used to report patients’ characteristics, the prevalence of PIMs and other medication issues identified, and results from the patient and implementer surveys. We plan to observe the direction of impact of medication review on PIMs and other medication issues identified. In addition, a paired t-test for the difference in two means will be used to determine any changes in PIMs before and after medication reviews, which could be used to estimate the effect size for sample size calculation during the next phase scale-up study.

#### Qualitative data

The survey findings will be supplemented with findings from the semi-structured in-depth interviews. The interviews will only be on a selected pool of implementers (physicians and pharmacists) from both hospitals and will be audio-recorded and transcribed for analysis. The transcripts will be coded using a hybrid approach of inductive and deductive coding. The codes will be examined and analysed for key themes.

### Data Management and protection

Informed consent will be taken from all participants involved either during the intervention or the evaluation phase of this study. All participants (patients, physicians, and pharmacists) will be given a unique subject identifier (ID), and the data collected will be anonymised. Informed consent forms, completed surveys, and audio recordings and transcripts from interviews will be kept in a locked cabinet, within an access-restricted office or stored on a secured network at the research sites. The findings from this study will be disseminated through platforms such as conferences and journal publications.

## Discussion

The Pro-M study aims to assess the feasibility of implementing a medication review in the outpatient clinic setting to facilitate appropriate prescribing for older adults with multimorbidity, as well as promoting communication between prescribers, pharmacists, and other physicians. Although effectiveness is not the primary focus of this study, we intend to collect indicators such as pre-post PIMs identified and any reduction in the number of PIMs to shed some light on the direction of the effect of the intervention. Overall, the findings from this study will not only inform feasibility of the intervention in the outpatient clinics, but also be used to inform any adaptations in the intervention or the process that might be needed when planning for the next phase scale-up study. This will be done mainly through data collected on patient recruitment rate, PIMs reduction, patients’ experiences during the intervention, cost-analysis, and feedback from the onsite implementation team. As this is a feasibility study, we want to first determine how to implement the workflow in a busy clinic, with the goal to upscale the intervention in phase 3, and the eventual goal to integrate this intervention as routine care for older adults with multimorbidity. In addition, most of the prescribing-related interventions are implemented in primary care, nursing homes and inpatient settings [38].Thus, our study will contribute to the existing literature by reporting the implementation of such an intervention in hospital outpatient settings and seek to inform hospital providers in Singapore and elsewhere who are looking to optimise prescribing in their settings.

The strength of conducting a feasibility study comes from the exploration of the implementation process in a smaller group that allows for adaptations in parameters like mode of recruitment or eligible criteria [39].In addition, the two sites involved have operational differences in their implementation, which will allow for observation of the same intervention elements in different contexts. Understanding the circumstances which something worked or did not work will inform future adaptions to other outpatient clinics.

There are also practical limitations to this study. Patients are recruited based on convenience sampling through referral by their attending physicians. Due to the small sample size, our results will not have adequate statistical power to detect significant change. However, demonstrating effectiveness is not an aim of this study. Thus, a larger scale-up study will be needed to determine its effectiveness and generalisability to older adults with multimorbidity in Singapore. Due to resource constraints, we are unable to recruit Tamil-speaking patients, which is also one of the official languages spoken in Singapore.

In conclusion, the Pro-M study will provide data on the feasibility of implementing an intervention to improve prescribing for older adults with multimorbidity in the outpatient clinics of public acute hospitals in Singapore. Although operational challenges are anticipated, results from the study will inform the feasibility of implementing this intervention on a wider scale in the future, with the potential to benefit older adults with multimorbidity.

### Electronic supplementary material

Below is the link to the electronic supplementary material.

Additional file 1: SPIRIT 2013 Checklist

Additional file 2: Pro-M patient survey

Additional file 3: Pro-M implementer survey

## Data Availability

Data sharing is not applicable to this article as no datasets were generated or analysed during the current study.

## Declarations

### Study status

At the time of submission, recruitment is ongoing for the study.

## Abbreviations

BCW: Behaviour Change Wheel
BCT: Behaviour Change Techniques
GRM: Geriatric Medicine
TDF: Theoretical Domains Framework
START: Screening Tool to Alert to Right Treatment
STOPP: Screening Tool of Older People’s Prescriptions
PIP: Potentially inappropriate prescribing
PIMs: Potentially inappropriate medications
