# Supplementary material for: Appropriate Prescribing for older adults with Multimorbidity (Pro-M): protocol for a feasibility study
Source: Arch Public Health. 2024 Mar 18;82:37. doi: 10.1186/s13690-024-01264-x (PMC10949664; doi:10.1186/s13690-024-01264-x)
Supplement: Supplementary file 2 — Additional file 2: Pro-M patient survey [file 13690_2024_1264_MOESM2_ESM.docx]

**PIP FEASIBILITY STUDY: PATIENT SURVEY**

This form is to collect data on patient experience for the feasibility study to implement an intervention to facilitate appropriate prescribing. It is to be administered by the research assistant/officer to the patient or caregiver. In the event that the patient is deemed cognitively impaired or unable to provide responses, the survey should be administered to the legal representative or caregiver.

| **Date:** |  |
| --- | --- |
| **Subject ID** |  |
| **Session attended by** | Patient alone  With legal representative/caregiver  (Relationship: ) |
| **Survey administered to** | Patient  Legal representative/caregiver |
| **Time taken to complete survey** |  |
| **Name of survey administrator** |  |

| **S/N** | **Survey items** | **Responses (1: Disagree/2: Somewhat disagree/3: Neutral/4: Somewhat agree/5: Agree)** |
| --- | --- | --- |
| **1** | The process of making an appointment for medication review was easy. | \| **1** \| **2** \| **3** \| **4** \| **5** \| \| --- \| --- \| --- \| --- \| --- \| |
| Comments: | | |
| **2** | I am willing to attend a separate appointment for the medication review. | \| **1** \| **2** \| **3** \| **4** \| **5** \| \| --- \| --- \| --- \| --- \| --- \| |
| Comments: | | |
| **3** | The purpose of the medication review was explained clearly to me by the doctor. | \| **1** \| **2** \| **3** \| **4** \| **5** \| \| --- \| --- \| --- \| --- \| --- \| |
| Comments: | | |
| **4** | My opinions on my medications were sought by either the pharmacist or doctor during this process. | \| **1** \| **2** \| **3** \| **4** \| **5** \| \| --- \| --- \| --- \| --- \| --- \| |
| Comments: | | |
| **5** | The doctor explained outcomes of the medication review to me in a way that I can understand. | \| **1** \| **2** \| **3** \| **4** \| **5** \| \| --- \| --- \| --- \| --- \| --- \| |
| Comments: | | |
| **6** | The medication review is a useful service for me. | \| **1** \| **2** \| **3** \| **4** \| **5** \| \| --- \| --- \| --- \| --- \| --- \| |
| Comments: | | |
| **7** | Overall, I am satisfied with the medication review service. | \| **1** \| **2** \| **3** \| **4** \| **5** \| \| --- \| --- \| --- \| --- \| --- \| |
| Comments: | | |
| **8** | I would recommend this service to other older adults with multiple medications or conditions. | \| **1** \| **2** \| **3** \| **4** \| **5** \| \| --- \| --- \| --- \| --- \| --- \| |
| Comments: | | |
| **9** | I would be willing to pay for the medication review service. | \| **1** \| **2** \| **3** \| **4** \| **5** \| \| --- \| --- \| --- \| --- \| --- \| |
| Comments: | | |
| **10** | I would be willing to pay up to $____ for the medication review service. | **$ __________** |
| Comments: | | |
| **11** | I would be willing to attend medication reviews when my doctor recommends me to do so. | \| **1** \| **2** \| **3** \| **4** \| **5** \| \| --- \| --- \| --- \| --- \| --- \| |
| Comments: | | |

-------- End of form --------
