# Supplementary material for: Appropriate Prescribing for older adults with Multimorbidity (Pro-M): protocol for a feasibility study
Source: Arch Public Health. 2024 Mar 18;82:37. doi: 10.1186/s13690-024-01264-x (PMC10949664; doi:10.1186/s13690-024-01264-x)
Supplement: Supplementary file 3 — Additional file 3: Pro-M implementer survey [file 13690_2024_1264_MOESM3_ESM.docx]

**Evaluation Survey for Implementers**

Thank you for participating in the evaluation that will inform the implementation process of the study. Your assessment will help us understand your experiences during the study, and how you perceive the impact of Pro-M on your clinical work, clinic work flow, patient care, and sustainability of medication review in routine outpatient care. Each statement contains a comment box, please feel free to comment on the statements, if elaboration is needed.

There are three sections in this evaluation form:

- Section 1 on demographic information to be answered by all
- Section 2 to be answered by those who delivered the intervention
- Section 3 to be answered by those who supported implementation of the study.
- Depending on the role you had in the study, you might need to answer both sections 2 and 3.
- Please note that some of you will be invited to attend an in-depth individual or group interview at a later date.

**Section 1: Demographics**

1. ❒ Site A ❒ Site B
2. ❒ Doctor ❒ Pharmacist
3. **Gender:**  ❒ Female ❒ Male
4. **For doctor: How long have you been practising in Geriatric Medicine? Month(s)**______ **Year(s)**
5. **For pharmacist: How long have you been practising as a pharmacist? Month(s)**______ **Year(s)**
6. **Please check the role(s) you played in the study:**

Delivery of intervention: Please complete Section 2

☐Supporting implementation of the study: Please complete Section 3

**Section 2: Delivery of intervention (Doctors and Pharmacists)**

|  | **Strongly disagree** | **Disagree** | **Neutral** | **Agree** | **Strongly**  **agree** | **Not applicable** |
| --- | --- | --- | --- | --- | --- | --- |
| 1. Suitable patients were easily identified using the inclusion criteria | 1 | 2 | 3 | 4 | 5 |  |
| Comment(s): | | | | | | |
| 1. Most of the eligible patients agreed to join the study **(Only applicable to doctors)** | 1 | 2 | 3 | 4 | 5 |  |
| Comment(s): | | | | | | |

What are the reasons given by patients/caregivers for not wanting to join the study? **(Only applicable to doctors)**

|  |
| --- |
|  |

|  | **Strongly disagree** | **Disagree** | **Neutral** | **Agree** | **Strongly agree** | **Not applicable** | |
| --- | --- | --- | --- | --- | --- | --- | --- |
| 1. The intervention did not disrupt my clinical workflow | 1 | 2 | 3 | 4 | 5 |  | |
| Comment(s): | | | | | | | |
| 1. The process to identify suitable patients for medication review was feasible | 1 | 2 | 3 | 4 | 5 |  | |
| Comment(s): | | | | | | | |
| 1. Memo/discussion on medication review findings is a good way of providing feedback to doctors | 1 | 2 | 3 | 4 | 5 |  | |
| Comment(s): | | | | | | | |
| 1. Collaboration between doctors and pharmacist on prescribing for older adults with multimorbidity is an acceptable practice | 1 | 2 | 3 | 4 | 5 |  | |
| Comment(s): | | | | | | | |
| 1. Discussions between doctors and pharmacists facilitated the collaboration | 1 | 2 | 3 | 4 | 5 |  | |
| Comment(s): | | | | | | | |
| 1. Documenting the MR results/prescribing decision with medication indication was feasible | 1 | 2 | 3 | 4 | 5 |  | |
| Comment(s): | | | | | | | |
| 1. Communicating changes made to other prescribers within my hospital was feasible **(Only applicable to doctors)** | 1 | 2 | 3 | 4 | 5 |  | |
| Comment(s): | | | | | | | |
| 1. Communicating changes made to external prescribers was feasible **(Only applicable to doctors)** | 1 | 2 | 3 | 4 | 5 |  | |
| Comment(s): | | | | | | | |
| 1. Feedback from the medication reviews facilitated my prescribing decisions **(Only applicable to doctors)** | 1 | 2 | 3 | 4 | 5 | |  |
| Comment(s): | | | | | | | |
| 1. I felt supported by the inputs from pharmacists to make necessary changes **(Only applicable to doctors)** | 1 | 2 | 3 | 4 | 5 | |  |
| Comment(s): | | | | | | | |
| 1. The tool of choice for medication review is effective in identifying potentially inappropriate medications | 1 | 2 | 3 | 4 | 5 | |  |
| Comment(s): | | | | | | | |
| 1. We should continue using a tool to identify potentially inappropriate medications | 1 | 2 | 3 | 4 | 5 | |  |
| Comment(s): | | | | | | | |
| 1. This medication review process could be integrated into routine care at the clinic | 1 | 2 | 3 | 4 | 5 | |  |
| Comment(s): | | | | | | | |

1. What challenges did you face during this study?

|  |
| --- |
|  |
|  |

1. Do you have any suggestions for improvement?

|  |
| --- |
|  |
|  |

**Section 3: Supporting implementation of the study (Doctors and Pharmacists)**

|  | **Strongly disagree** | | | **Disagree** | | | **Neutral** | | | **Agree** | | | **Strongly agree** | | | | | **Not applicable** |
| --- | --- | --- | --- | --- | --- | --- | --- | --- | --- | --- | --- | --- | --- | --- | --- | --- | --- | --- |
| 1. Suitable patients were easily identified using the inclusion criteria. | 1 | | | 2 | | | 3 | | | 4 | | | 5 | | | | |  |
| Comment(s): | | | | | | | | | | | | | | | | | | |
| 1. Suitable patients were easily recruited into the study | 1 | | | 2 | | | 3 | | | 4 | | | 5 | | | | |  |
| Comment(s): | | | | | | | | | | | | | | | | | | |
| 1. The recruitment process was adapted to increase patients’ participation | | | 1 | | | 2 | | | 3 | | | 4 | | 5 | |  | | |
| Comment(s): | | | | | | | | | | | | | | | | | | |
| 1. The intervention did not disrupt usual clinical workflow | | | 1 | | | 2 | | | 3 | | | 4 | | 5 | |  | | |
| Comment(s): | | | | | | | | | | | | | | | | | | |
| 1. The intervention workflow was adhered to: Pharmacist communicates MR results to doctor > Doctor reviews MR results and makes prescribing decisions > Doctor communicates decisions to other prescribers when needed | | | 1 | | | 2 | | | 3 | | | 4 | | 5 | | |  | |
| Comment(s): | | | | | | | | | | | | | | | | | | |
| 1. Timely feedback from doctors/pharmacists/clinic staff was provided to improve the intervention process | | | 1 | | | 2 | | | 3 | | | 4 | | 5 | |  | | |
| Comment(s): | | | | | | | | | | | | | | | | | | |
| 1. The protocol training helped doctors and pharmacists to carry out the intervention accordingly | | | 1 | | | 2 | | | 3 | | | 4 | | 5 | |  | | |
| Comment(s): | | | | | | | | | | | | | | | | | | |
| 1. Information/data were updated into the system in a timely manner | | | 1 | | | 2 | | | 3 | | | 4 | | 5 | |  | | |
| Comment(s): | | | | | | | | | | | | | | | | | | |
| 1. Issues/problems were reported and resolved in a timely manner | | | 1 | | | 2 | | | 3 | | | 4 | | 5 | |  | | |
| Comment(s): | | | | | | | | | | | | | | | | | | |
| 1. This medication review process can be integrated into routine care at the clinic | | 1 | | | 2 | | | 3 | | | 4 | | | | 5 | | |  |
| Comment(s): | | | | | | | | | | | | | | | | | | |

1. What challenges did you face during this study?

|  |
| --- |
|  |
|  |

1. Do you have any suggestions for improvement?

|  |
| --- |
|  |
|  |
